# Supplementary material for: Coherence transfer from optically induced THz magnons to charges
Source: Nat Commun. 2026 Feb 6;17:1480. doi: 10.1038/s41467-026-69261-y (PMC12886983; doi:10.1038/s41467-026-69261-y)
Supplement: Supplementary file 1 — Supplementary Information [file 41467_2026_69261_MOESM1_ESM.pdf]

Supplementary Information of
"Coherence transfer from optically induced THz magnons to charges"

Moritz Cimander[1], Volker Wiechert[1], Julian Bär[1], Takuya Satoh[2], Jörg Bünemann[3], Götz S. Uhrig[3] and Davide Bossini[1]*

[1]Department of Physics and Center for Applied Photonics, University of Konstanz, D-78457 Konstanz, Germany

[2]Department of Physics, Institute of Science Tokyo, Tokyo 152-8551, Japan

[3]Condensed Matter Theory, TU Dortmund University, Otto-Hahn-Straße 4, Dortmund 44221, Germany

*Corresponding author. Email: davide.bossini@uni-konstanz.de.

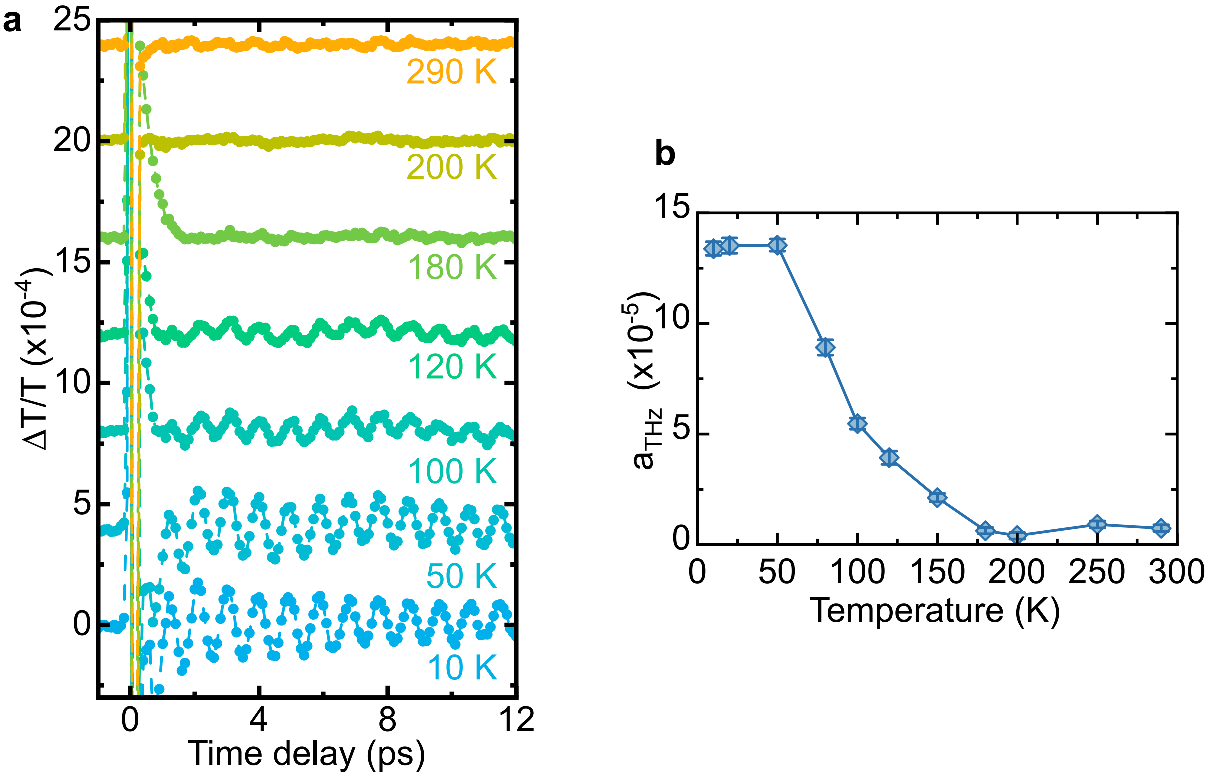

Supplementary Fig. 1 Temperature dependence of the transient transmissivity. a, Transient transmissivity for different values of the sample temperature. The pump and the probe beams are horizontally linearly polarized. The pump fluence is 24 mJ/cm^2 and the spectrum is centered around 0.98 eV. The probe central photon energy is 1.65 eV. The temperature is ramped up from the lowest to the highest value. **b** Temperature dependence of the amplitudes a_{THz} of the THz oscillations in panel **a**. The amplitudes were obtained by fitting the data in panel **a** with the function shown in Eq. (M1). The error bars are given by the standard error of the amplitudes obtained by fitting the data in the time-domain. The standard error is calculated from the width of 95% confidence interval.

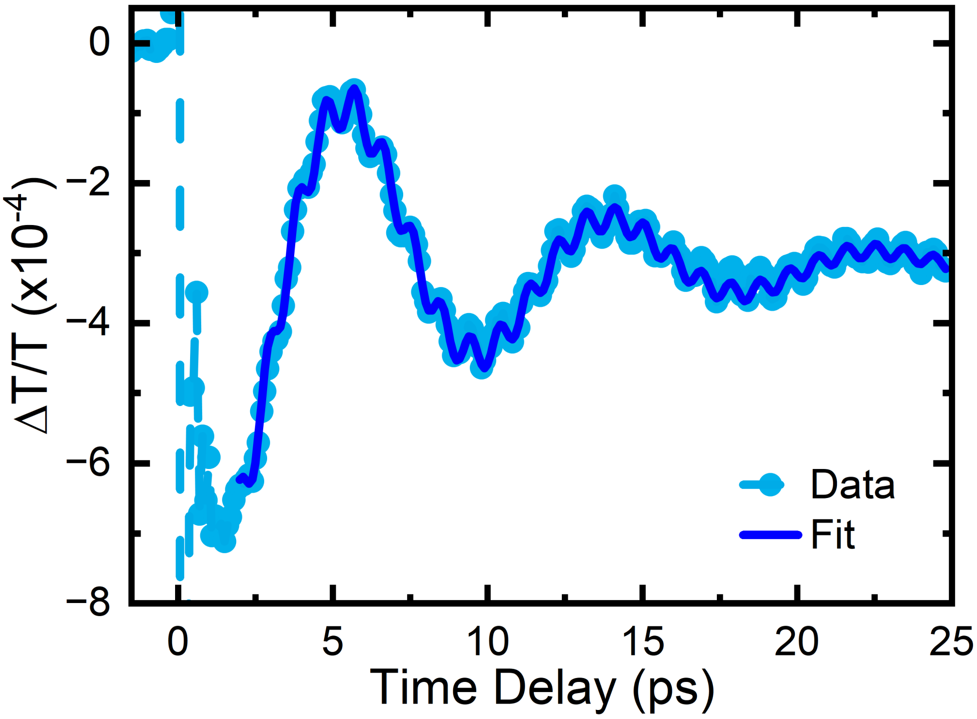

Supplementary Fig. 2 Fitting of the transient transmissivity. Transient transmissivity detected with a probe beam with photon energy of 1.65 eV and a linear polarization along the vertical axis. The pump pulses are linearly horizontally polarized, with a fluence of 24 mJ/cm^2 and a central photon energy of 0.98 eV. The sample temperature is set to 10 K. The dark blue line shows the fitted curve, which is derived from the procedure described in the Methods (Eq. (M1)).

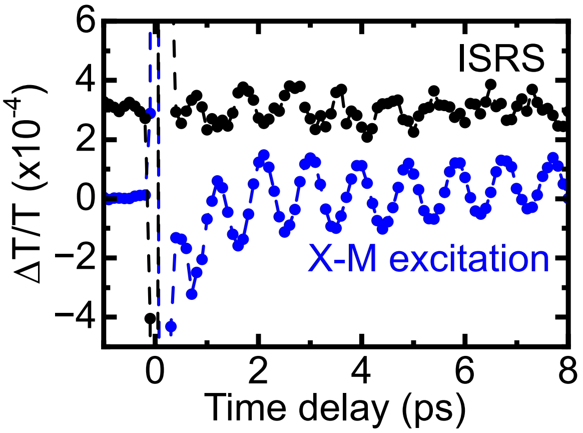

Supplementary Fig. 3 Resonant vs. non-resonant excitation. The non-resonant excitation (black data points) was performed with pump pulses centered around 1.1 eV, fluence of 24 mJ/cm^2 and a linear polarization rotated by 120° relative to the horizontal plane. The blue data points depict the THz signal, which is observable for resonant pumping. In this case the pump beam has a central photon energy of 0.98 eV and is linearly polarized in the horizontal plane. The fluence was set to 24 mJ/cm^2. Both experiments have the same probe beam configuration. The probe beam is centered around 1.65 eV and is polarized in the horizontal plane.
